# The Road to Tuberculosis (*Mycobacterium tuberculosis*) Elimination in Arkansas; a Re-Examination of Risk Groups

**DOI:** 10.1371/journal.pone.0090664

**Published:** 2014-03-11

**Authors:** Anna Berzkalns, Joseph Bates, Wen Ye, Leonard Mukasa, Anne Marie France, Naveen Patil, Zhenhua Yang

**Affiliations:** 1 Department of Epidemiology, School of Public Health, University of Michigan, Ann Arbor, Michigan, United States of America; 2 Arkansas Department of Health, Little Rock, Arkansas, United States of America; 3 Department of Epidemiology, Fay W. Boozman College of Public Health, University of Arkansas for Medical Sciences, Little Rock, Arkansas, United States of America; 4 Department of Biostatistics, School of Public Health, University of Michigan, Ann Arbor, Michigan, United States of America; 5 Division of Tuberculosis Elimination, Centers for Disease Control and Prevention, Atlanta, Georgia, United States of America; 6 College of Medicine, University of Arkansas for Medical Sciences, Little Rock, Arkansas, United States of America; Public Health Agency of Barcelona, Spain

## Abstract

**Objectives:**

This study was conducted to generate knowledge useful for developing public health interventions for more effective tuberculosis control in Arkansas.

**Methods:**

The study population included 429 culture-confirmed reported cases (January 1, 2004–December 31, 2010). *Mycobacterium tuberculosis* genotyping data were used to identify cases likely due to recent transmission (clustered) versus reactivation (non-clustered). Poisson regression models estimated average decline rate in incidence over time and assessed the significance of differences between subpopulations. A multinomial logistic model examined differences between clustered and non-clustered incidence.

**Results:**

A significant average annual percent decline was found for the overall incidence of culture-confirmed (9%; 95% CI: 5.5%, 16.9%), clustered (6%; 95% CI: 0.5%, 11.6%), and non-clustered tuberculosis cases (12%; 95% CI: 7.6%, 15.9%). However, declines varied among demographic groups. Significant declines in clustered incidence were only observed in males, non-Hispanic blacks, 65 years and older, and the rural population.

**Conclusions:**

These findings suggest that the Arkansas tuberculosis control program must target both traditional and non-traditional risk groups for successful tuberculosis elimination. The present study also demonstrates that a thorough analysis of TB trends in different population subgroups of a given geographic region or state can lead to the identification of non-traditional risk factors for TB transmission. Similar studies in other low incidence populations would provide beneficial data for how to control and eventually eliminate TB in the U.S.

## Introduction

Following the 1992 peak in tuberculosis (TB) incidence in the United States, the annual TB incidence rate has decreased every year [1]. In 2011, the incidence was 3.4 cases per 100,000 persons, the lowest recorded rate since national TB reporting began in 1953 [2]. However, this rate failed to meet the national goal of TB elimination by 2010, defined as ≤1 case per million persons [2]. TB case rates differ between sexes, among age, racial/ethnic groups, and geographic regions [1], [3]. Both reactivation of latent TB infection (LTBI) and recent transmission contribute to the overall disease burden in the U.S. Understanding incidence trends and the relative contributions of reactivation and recent transmission to the disease burden in subpopulations can maximize the use of limited resources available for TB elimination programs.

Molecular epidemiological studies can help characterize TB transmission [4], [5]. Variation in the *Mycobacterium tuberculosis* (MTB) genome allows genotyping to differentiate among strains and identify cases that are more likely to be related by transmission [6]. Currently, the Centers for Disease Control and Prevention (CDC) supports TB genotyping of at least one MTB isolate from each culture-positive case in the U.S. through the National Tuberculosis Genotyping Service (NTGS) [7]. MTB isolates submitted to NTGS are genotyped using two methods: spacer oligonucleotide typing (spoligotyping) and mycobacterial interspersed repetitive unit-variable number tandem repeat typing (MIRU-VNTR). Cases with indistinguishable isolate genotypes are generally considered part of the same chain of transmission, when having either close geographic proximity or epidemiologic link [6]. Cases with a TB genotype that match that of at least one other case are referred to as clustered. However, as suggested by an earlier molecular epidemiological study conducted in Arkansas, while genotyping clustering provides an estimate of recent transmission, it is not a definitive measure [8]. Thus, a time-restricted cluster definition has been applied in molecular epidemiological studies of TB to improve the accuracy of genotyping clustering in measuring recent TB transmission [9], [10]. If clustered cases are diagnosed within a short period of time within the same geographic area, then recent transmission is more likely. Cases having unique isolates are more likely due to the reactivation of remotely acquired infection [9]. In the U.S., most molecular epidemiology TB studies have focused on large urban areas with higher TB incidence. However, recent studies have included rural areas and low-incidence populations, which have provided new insights into TB epidemiology [11]–[13]. Different forces may drive TB incidence between rural and urban populations and a better characterization of these forces should lead to better TB control.

TB incidence in Arkansas is relatively low and the case rate declined from 7.9 in 1997 to 2.7 in 2010 [1], [14]. However, recently the decline began to plateau, only decreasing by 0.2 cases per 100,000 during 2008–2010. Comparatively, during 2004–2006 incidence declined 1.2 cases per 100,000 and during 2006–2008 the decline was 0.7 cases per 100,000 [1], [15], [16]. Understanding the trends in TB due to either recent transmission or reactivation of latent infection in different subpopulations in Arkansas is essential to successfully target control efforts. Characterizing these trends will aid in understanding which subpopulations are contributing to the decline.

France and coworkers analyzed TB trends in Arkansas during 1997–2003 [10]. Non-Hispanic blacks and individuals aged 65 years or greater had the greatest overall declines in incidence. However, in both subgroups, incidence among non-clustered cases declined the most, suggesting that the primary driver behind the decline was a decrease in the rate of reactivation of past infections [10]. The overall TB incidence trend has changed since 2003, especially as the rate of the decline began to decrease in 2008.

Few studies have focused on understanding TB trends in low-incidence, rural populations [10]–[13]. In 2010, 35 states (70%) had incidence of less than 3.5 cases per 100,000 persons, the rate established in 1989 as the year 2000 interim target for TB elimination [1], [3]. As the number of low-incidence states increases, understanding incidence trends and factors driving the trends in these populations is vital for achieving TB elimination in the U.S. This study successfully analyzed TB incidence trends in Arkansas during 2004–2010 and the incidence trends in subpopulations based on previously identified TB risk factors, including age [17], [18], sex [19], geographic region [19], [20], race/ethnicity [21], and country of origin [22] in order to explore recent changes in incidence in these groups and evaluate the impact of recent transmission.

## Methods

### Study population and data sources

The study sample included culture-confirmed TB cases diagnosed in Arkansas during January 1, 2004–December 31, 2010 for which genotype data (spoligotype and 12-locus MIRU-VNTR results) were available [7]. Only the first isolate collected for each case was included in the analysis. Demographic and clinical information was obtained from a de-identified Arkansas TB surveillance database that is based on information collected by the Arkansas Department of Health using the CDC's “Report of a Verified Case of Tuberculosis” form. Information from the 2010 U.S. Census was used to describe the demographics of the population of Arkansas [23]. Urban and rural areas of Arkansas were defined using methods previously described, based on the Census Bureau Metropolitan Statistical Areas (MSAs) defined by the Office of Management and Budget [10], [24]. The study was approved by the Institutional Review Board for Health Science and Behavior Science of the University of Michigan.

### Incidence rate calculations

State TB case rates for culture-confirmed, clustered, and non-clustered TB were calculated from TB surveillance data provided by the Arkansas Department of Health. The annual TB case rates for the total population and each subpopulation were calculated using the July 1^st^ population estimate for each year, obtained from the Bridged-Race Population Estimates provided by the National Center for Health Statistics [25]. The data for the foreign born population were obtained from the American Community Survey [26].

### Cluster definition

The study patients were classified as clustered and non-clustered cases, estimating TB due to recent transmission and reactivation of latent TB infection respectively, using a combination of genotype information of *M. tuberculosis* isolates and diagnostic dates of the study patients. A case was defined as clustered with another case in the state if the isolates of the two cases had an identical spoligotype and 12-locus MIRU-VNTR genotyping pattern and both cases were diagnosed within a one year time period [27]; a cluster may span more than one year when it involves more than two cases with identical spoligotype and 12-locus MIRU-VNTR genotyping pattern that were diagnosed in different years, but were connected to at least one other case within a one year time frame. When diagnosis date was not available, count date (date the patient was verified as a TB case by the health department) was used as a proxy. This cluster definition has been used previously to estimate recent transmission [10]. Isolates that did not meet this cluster definition were classified as non-clustered.

### Statistical Analysis

Chi-squared tests were used to compare the distribution of demographic and clinical factors between the culture-confirmed cases with genotype information and the culture-confirmed cases without genotype information, in order to assess the representativeness of the overall–culture confirmed TB patient population in the study sample. To visually examine the trend in incidence, the culture-confirmed annual incidence rate was plotted over time for each of the population subgroups. Confidence intervals were calculated based on a Poisson distribution, using a method described by Buchanan in Microsoft Excel [28].

A Poisson model was used to estimate the average percent decline in incidence of culture-confirmed TB over time. To examine whether clustered cases and non-clustered cases had a similar average annual percent decline in incidence during the study period, we used a multinomial logistic model, given that Poisson models do not allow us to directly compare the two types of cases. Since TB incidence is very low in Arkansas, the estimated relative risk ratio of clustered cases compared to non-clustered cases obtained from this model is approximately equal to the incidence ratio. Therefore we used this model to test the difference between the trends of clustered and non-clustered cases.

In addition, univariate Poisson regression was used to assess whether there was any difference in the trends of percentage decline in culture-confirmed, clustered, and non-clustered TB incidence over the study period between age groups (20 to 64 years vs. 65 and older), race/ethnic groups (non-Hispanic whites vs. non-Hispanic blacks), geographic regions (urban vs. rural counties), country of origin (foreign born vs. US born), and sexes, respectively. Analysis of these demographic factors was done one at a time. The age groups were chosen to allow for comparison to the previous Arkansas study. Cases less than 20 years of age were excluded from the analysis because the sample size is too small to allow for meaningful statistical analysis. For each analysis, the model included one demographic variable, the time variable, and the interaction between time and the demographic variable. If a regression coefficient of the interaction term is significantly different from zero, it indicates that the particular demographic variable is significantly associated with a percent decline in the rate of TB. Similar analyses were also done separately on clustered TB cases and non-clustered cases to estimate the association of the above demographic factors on the decline rate of recent transmission and reactivation, respectively. For each of the above regression analyses, we compared a linear time function and a quadratic time function of log of TB incidence rate. For all analyses, a model with the linear time function yielded a better fit for our data and therefore was used in all analyses. These statistical analyses were performed in SAS version 9.2 [29]. All analyses were completed using de-identified data. In all analyses, P-value<0.05 was considered to be statistically significant.

## Results

### Characteristics of the study population

A total of 699 cases of TB were reported in Arkansas during the study period (January 1, 2004–December 31, 2010). Of the total reported cases, 494 (70.7%) were confirmed by mycobacterial culture of sputum, tissue, or body fluids. Genotyping information was available for 429 (86.8%) of the 494 culture-confirmed TB cases. Most of the reported cases were either non-Hispanic white or non-Hispanic black (Table 1). A majority of the cases were male and in the 20 to 64 year age range. Almost 70% of the cases were born in the United States.

**Table 1:** Distribution of Demographic Characteristics, Risk Factors, and Clinical Characteristics Among All Tuberculosis Cases Reported in Arkansas During 2004–2010.

	No. of cases	%
**Total**	699	100
**Race/ethnicity**		
Non-Hispanic White	258	36.9
Non-Hispanic Black	207	29.6
Hispanic	122	17.5
Asian/Pacific Islander	91	13.0
American Indian/Alaskan Native	2	0.3
Unknown	19	2.7
**Age groups (years)**		
<20	94	13.4
20–64	410	58.7
65–84	165	23.6
≥85	30	4.3
**Sex**		
Male	431	61.7
Female	261	37.3
Unknown	7	1.0
**Place of birth**		
Foreign-born	211	30.2
US-born	488	69.8
**Geographic area**		
Rural	262	37.5
Urban	437	62.5
**Site of disease**		
Pulmonary	567	81.1
Extrapulmonary	92	13.2
Both	40	5.7

We included all 429 culture-confirmed cases with available genotyping information in our study sample. To assess the sample representativeness, we compared the distribution of patient characteristics among genotyped cases with that among all the non-genotyped culture-confirmed cases. The distributions of age group and site of disease (pulmonary vs. extrapulmonary) were significantly different (*P*<0.05) between genotyped and the non-genotyped culture-confirmed cases (Table 2). The distributions of sex, race/ethnicity, place of birth, and type of residential area were not significantly different between the genotyped and non-genotyped cases.

**Table 2:** Comparison of Distribution of Selected Demographic and Clinical Characteristics among the 429 Genotyped Tuberculosis Cases Included in the Study Sample with that among the 65 Culture-Confirmed Tuberculosis Cases Excluded from the Study due to Lack of Genotyping Information.

	Genotyped (n = 429), No. (%)	Not genotyped culture-confirmed (n = 65), No. (%)	*P*-value*
**Race/ethnicity** a			
Non-Hispanic White	174 (40.6)	28 (43.1)	0.9004
Non-Hispanic Black	140 (32.6)	22 (33.8)	
Other	106 (24.7)	15 (23.1)	
**Age groups (years)**			
<20	28 (6.5)	0 (0)	0.005
20–64	266 (62.0)	33 (50.8)	
65–84	115 (26.8)	23 (35.4)	
≥85	20 (4.7)	9 (13.8)	
**Sex** b			
Male	262 (61.1)	48 (73.8)	0.0993
Female	160 (37.3)	17 (26.2)	
**Place of birth**			
Foreign-born	110 (25.6)	12 (18.5)	0.1873
US-born	319 (74.4)	53 (81.5)	
**Geographic area**			
Rural	164 (38.2)	30 (46.2)	0.2691
Urban	265 (61.8)	35 (53.8)	
**Site of disease**			
Pulmonary	363 (84.6)	46 (70.8)	0.0237
Extrapulmonary	42 (9.8)	11 (16.9)	
Both	24 (5.6)	8 (12.3)	

*P- value from chi-square test.

a Race-ethnicity was unknown for 9 genotyped cases.

b Sex was unknown for 7 genotyped cases.

### Clustering of cases

Of the 429 genotyped cases, 178 (41.5%) were clustered and 251 (58.5%) were non-clustered. The clustered cases consisted of a total of 60 clusters, of which 47 (78.3%) had between two and four related cases and 7 (11.7%) clusters had between five and nine related cases. The remaining 6 (10%) clusters had 10 or more cases, with the largest cluster containing 20 cases. The time spans of clusters with 10 or more cases ranged from four years to the entire study period of seven years.

### State-wide incidence trends

**Figure 1:**
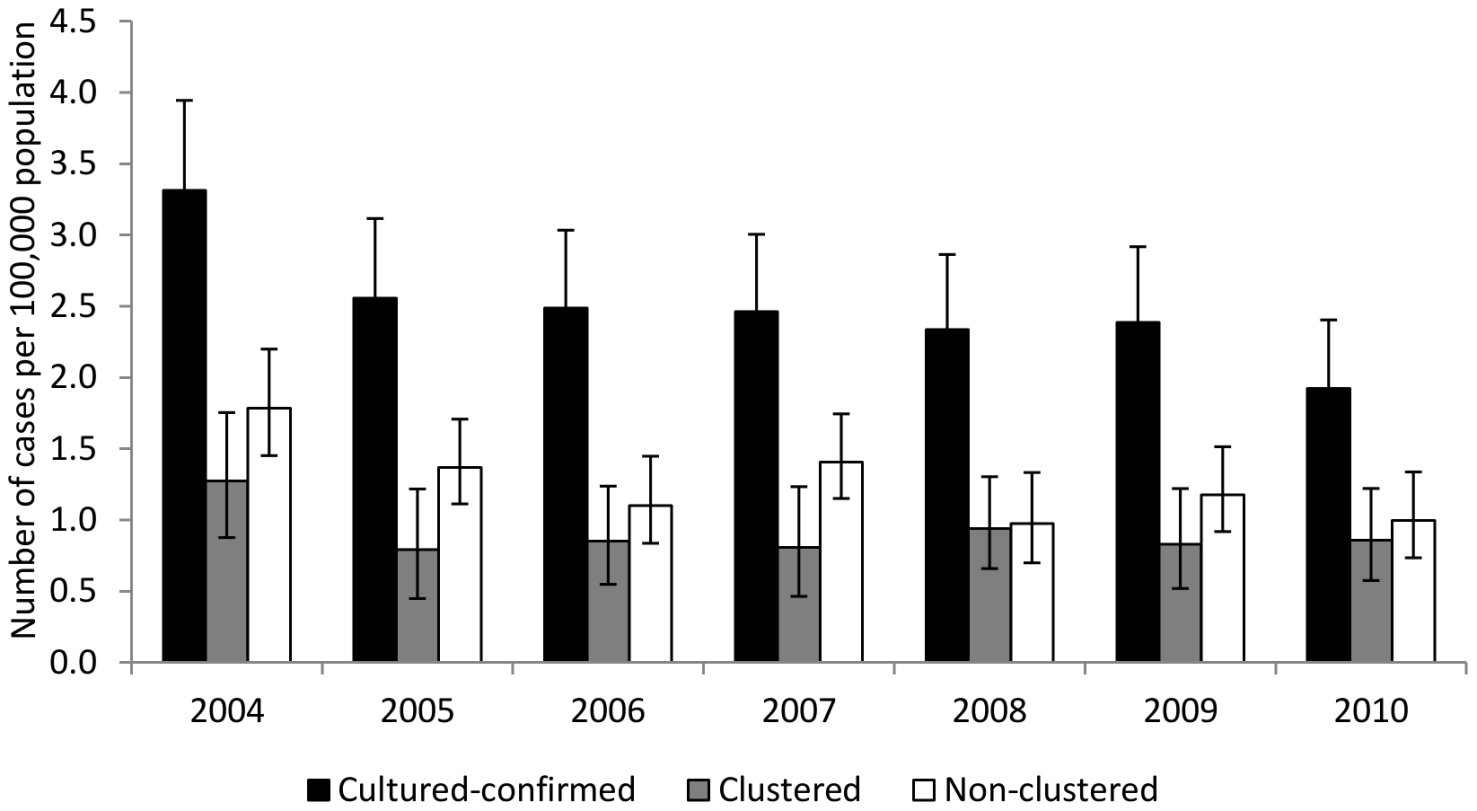
Estimated incidence of culture-confirmed, clustered, and non-clustered tuberculosis cases. Estimated incidence and 95% confidence intervals of culture-confirmed, clustered, and non-clustered cases of tuberculosis reported in Arkansas from 2004 to 2010. A case was included in a time-restricted cluster if it had identical spoligotype and 12 loci MIRU to another isolate diagnosed within the 1-year period prior to its diagnosis date.

During January 1, 2004–December 31, 2010, the annual incidence of culture-confirmed cases in Arkansas declined by 1.4 cases per 100,000 persons (Figure 1). However, during January 1, 2008–December 31, 2010 the annual incidence of such cases declined only by 0.2 per 100,000. During the study period, the annual incidence of clustered TB and non-clustered TB declined by 0.4 and 0.8 cases per 100,000, respectively (Figure 1). The average annual percent decline in incidence for culture-confirmed cases was approximately 9% (95% CI: 5.5%, 16.9%). Based on the results from the multinomial logistic model, the annual percent decline in incidence for the clustered cases and non-clustered cases was 6% (95% CI: 0.5%, 11.6%) and 12% (95% CI: 7.6%, 15.9%), respectively. Although the non-clustered TB experienced a higher average annual percent decline than clustered TB, the observed difference was not statistically significant (P = 0.11).

### Incidence trend comparison between age groups

**Figure 2:**
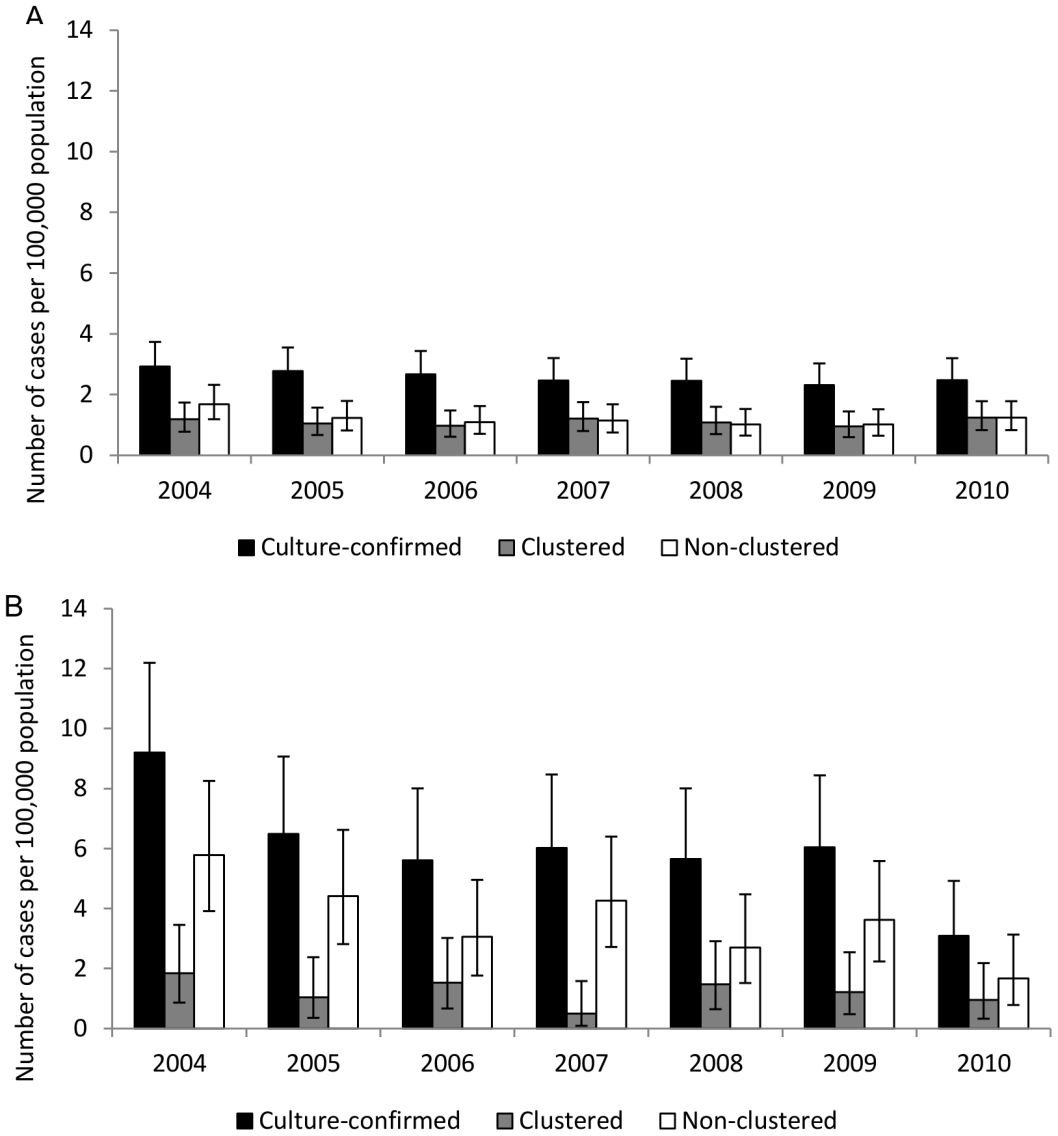
Comparison of culture-confirmed, clustered, and non-clustered tuberculosis cases by age group. Comparison of incidence trends of culture-confirmed, clustered, and non-clustered cases of tuberculosis reported in Arkansas from 2004 to 2010 between two major age groups. a: among individuals 20 to 64 years of age; b: among individuals 65 years or older.

The incidence of culture-confirmed TB was consistently higher in the older age group (≥65) than in the younger age group (20–64) during each year of the study period. However, a larger absolute decline in the annual incidence of culture-confirmed TB was observed in the ≥65 years age group (6.1 per 100,000) than in the 20–64 year age group (0.5 per 100,000) (Figure 2). In the older age group, clustered incidence declined by 0.8 per 100,000 and non-clustered declined by 4.1 per 100,000. No change in clustered TB was observed in the younger age group, while non-clustered declined by 0.5 per 100,000. The older age group (11.9%, 95% CI: 8.2%, 15.5%) experienced a significantly higher average annual percent decline in incidence than the younger age group (1.8%, 95% CI: −5.3%, 8.3%) in culture-confirmed TB (P = 0.0082). A similar trend was also observed in the clustered TB cases, the older age group (12.5%, 95% CI: 5.6%, 18.9%) experienced a significantly higher average annual percent decline (P = 0.0062) than the younger age group (−3.9%, 95% CI: −14.5%, 5.7%). In contrast, the average annual percent decline in incidence in the non-clustered cases was not significantly different between the two age groups (Table 3).

**Table 3:** Average Annual Percent Decline of Incidence from the Univariate Poisson Regression Models of Tuberculosis Incidence in Arkansas During 2004–2010 Testing the Effects of Time, Race/ethnicity, Sex, Age Group, and Geographic Area of Residence and Interactions of Time and Race/ethnicity, Time and Sex, Time and Age Group, and Time and Geographic Area.

		Culture-confirmed cases		Clustered cases		Non-clustered cases	
	Demographic groups	Average percent decline of incidence (95% CI)	P-value	Average percent decline of incidence (95% CI)	P-value	Average percent decline of incidence (95% CI)	P-value
Race	Non-Hispanic Black	14.5% (9.4, 19.4)	0.0067	12.9% (4.7, 20.3)	0.034	17.7% (10.2, 23.4)	0.065
	Non-Hispanic White	5.3% (0.9, 10.4)		0.8% (−7.5, 8.4)		9.0% (3.4, 14.2)	
Sex	Female	7.8% (2.7, 12.7)	0.58	3.9% (−5.7, 12.7)	0.53	10.2% (3.7, 16.3)	0.49
	Male	9.6% (5.2, 13.8)		7.6% (0.4, 14.7)		13.2% (7.4, 18.6)	
Age Group	≥65 years	11.9% (8.2, 15.5)	0.0082	12.5% (5.6, 18.9)	0.0062	13.8% (9.2, 25.7)	0.15
	20 to 64 years	1.8% (−5.3, 8.3)		−3.9% (−14.5, 5.7)		5.6% (−5.6, 15.6)	
Geographic Area	Rural	10.0% (5.2, 14.7)	0.47	9.8% (1.2, 17.7)	0.26	11.4% (5.2, 21.5)	0.87
	Urban	7.7% (3.0, 12.1)		3.4% (−4.5, 11.3)		12.1% (6.1, 17.8)	
Country of origin	Foreign	15.6% (9.6, 28.8)	0.022	12.9% (−2.7, 26.1)	0.21	7.5% (−3.6, 17.5)	0.85
	U.S. born	7.2% (3.0, 11.2)		2.0% (−6.3, 9.7)		8.7% (1.6, 15.3)	

### Incidence trend comparison between sexes

**Figure 3:**
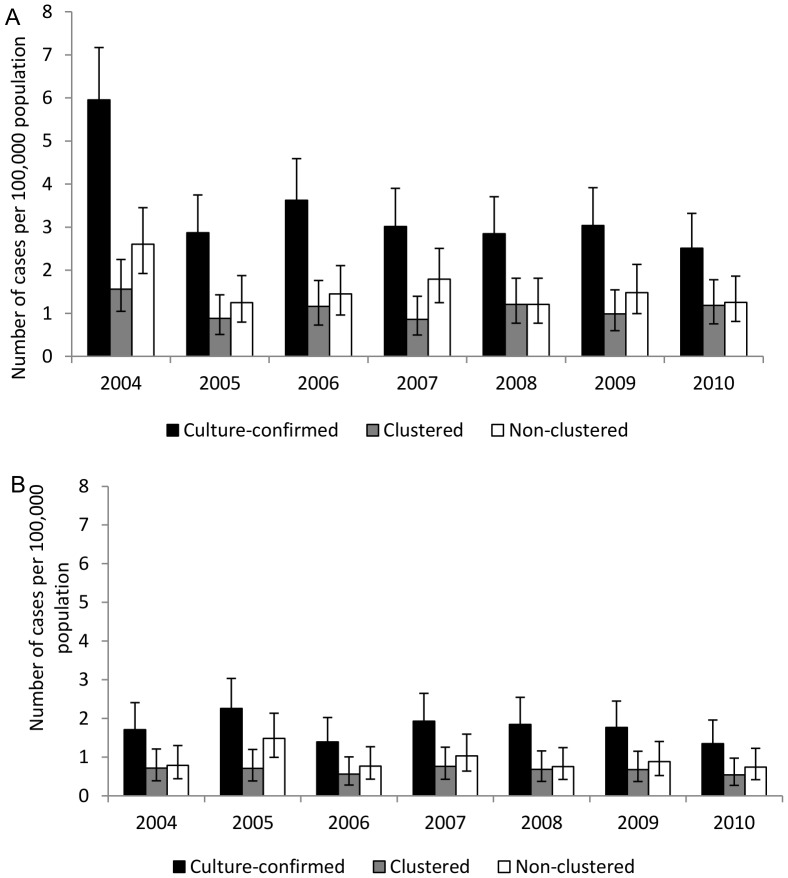
Comparison of culture-confirmed, clustered, and non-clustered tuberculosis cases by sex. Comparison of incidence trends of culture-confirmed, clustered, and non-clustered cases of tuberculosis reported in Arkansas from 2004 to 2010 between two sexes. a: among males; b: among females.

The incidence of culture-confirmed TB was consistently higher in males than females over the study period (Figure 3). Males experienced a larger absolute overall decline in the culture-confirmed TB incidence than females (3.5 vs. 0.4 per 100,000). A larger decline was observed in non-clustered TB (1.3 per 100,000) than in clustered TB (0.4 per 100,000) among males. However, among females the decline was similar, with non-clustered declining by 0.1 per 100,000 and clustered declining by 0.2 per 100.000. Despite these differences, the average annual percent decline in incidence in culture-confirmed, clustered, or non-clustered TB was similar between females and males (Table 3).

### Incidence trend comparison between races/ethnicities

**Figure 4:**
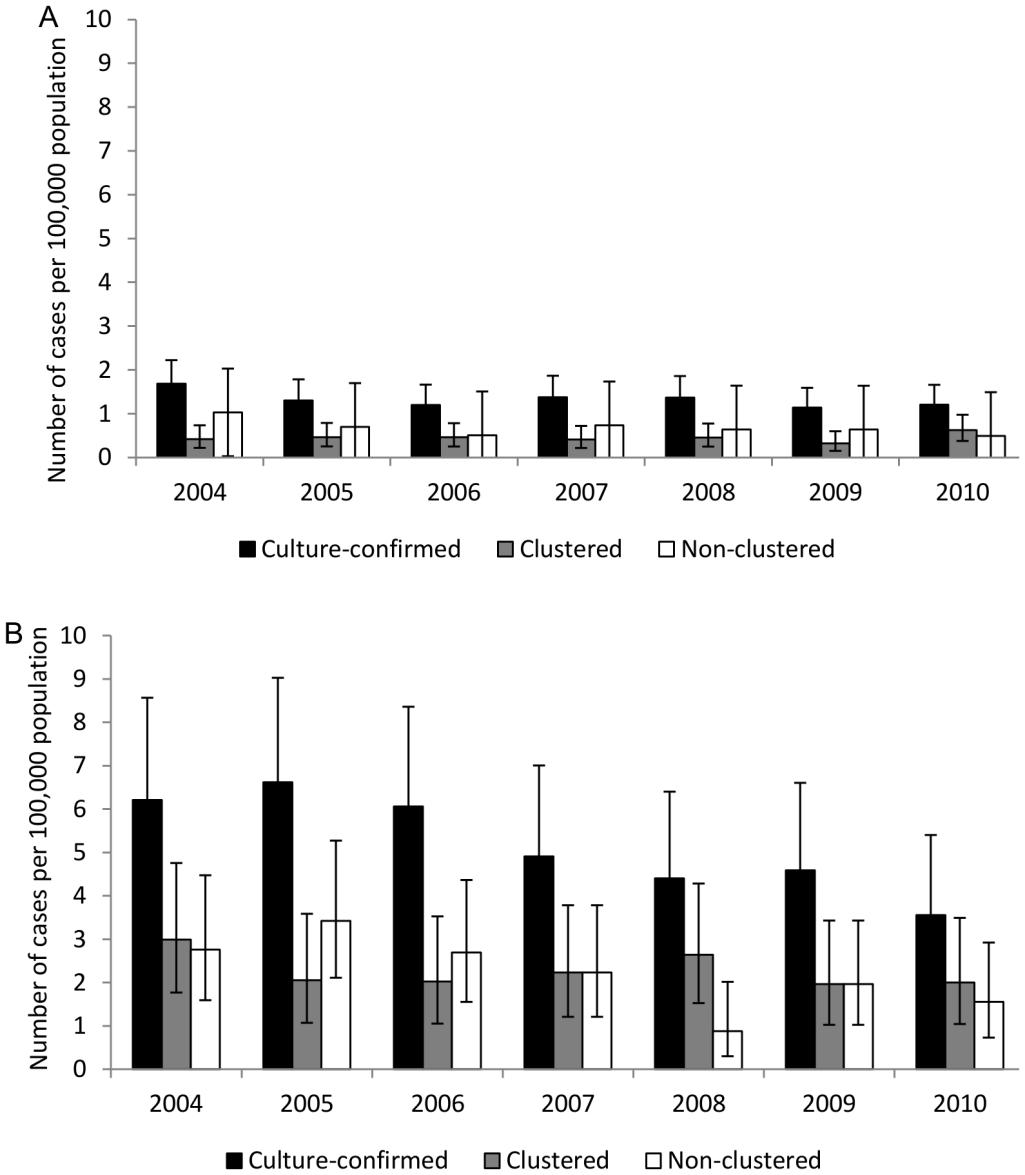
Comparison of culture-confirmed, clustered, and non-clustered tuberculosis cases by race/ethnicity. Comparison of incidence trends of culture-confirmed, clustered, and non-clustered cases of tuberculosis reported in Arkansas from 2004 to 2010 between two major race/ethnic groups. a: among non-Hispanic whites; b: among non-Hispanic blacks.

Across the entire study period, the annual incidence of culture-confirmed TB was lower for non-Hispanic whites than for non-Hispanic blacks (Figure 4). The absolute decline in the incidence of culture-confirmed TB was 0.5 and 2.6 per 100,000 for non-Hispanic whites and non-Hispanic blacks, respectively. Among non-Hispanic whites, non-clustered TB declined by 0.5 per 100,000 while clustered TB increased by 0.2 cases per 100,000. Among non-Hispanic blacks, the declines in non-clustered TB (1.2 per 100,000) and clustered TB (1.0 per 100,000) were similar. The average annual percent decline in incidence for culture-confirmed TB was significantly higher (P = 0.0067) in non-Hispanic blacks (14.5%, 95% CI: 9.4%, 19.4%) than in non-Hispanic whites (5.3%, 95% CI: 0.9%, 10.4%). A statistically significant (P = 0.034) difference in the incidence trend of clustered TB was also found between the groups (Table 3). The annual incidence of clustered TB in non-Hispanic whites showed little change, with an average annual percent decline in incidence of 0.8% (95% CI: −7.5%, 8.4%) while it declined significantly in non-Hispanic blacks, with an average annual percent decline in incidence of 12.9% (95% CI: 4.7%, 20.3%). In contrast, the incidence of non-clustered TB declined significantly in both races/ethnicities with no significant different between them (Table 3).

### Incidence trend comparison between urban and rural areas

The incidence rates of culture-confirmed TB in individuals from counties classified as rural and individuals from urban counties were similar over the study time period. The absolute decline in overall culture-confirmed TB incidence was 2.0 and 1.0 per 100,000 persons in the rural population and the urban population, respectively. Clustered TB declined by 0.1 per 100,000 and non-clustered TB declined by 0.8 per 100,000 in the urban population. Among rural residents, the declines were similar with clustered declining by 0.9 per 100,000 and non-clustered by 0.8 per 100,000. The average annual percent decline in incidence of all the culture-confirmed cases, whether clustered or non-clustered TB, was not significantly different between rural and urban residents (Table 3).

### Incidence trend comparison between the U.S. born and the foreign born

Over the study period, the incidence of culture-confirmed TB in the foreign born was greater than in U.S. born individuals. However, in the foreign born population, culture-confirmed TB declined by 30.2 per 100,000 persons while in the U.S. born population, the decline was 1.1 per 100,000 persons. In the foreign born population, clustered TB declined by 8.4 per 100,000 persons and non-clustered by 5.7 per 100,000. In the U.S. born population, the decline in clustered incidence was 0.8 per 100,000 persons and 0.7 per 100,000 in non-clustered cases. The average annual percent decline in incidence of culture-confirmed TB was significantly higher (P = 0.022) in the foreign born individuals (15.6%, 95% CI: 9.6%, 28.8%) than in the U.S. born individuals (7.2%, 95% CI: 3.0%, 11.2%). The average annual percent decline in the incidence of clustered TB was higher among foreign born individuals compared to U.S. born individuals; however, the observed difference was not statistically significant (Table 3). The average annual percent decline in the incidence of non-clustered TB was also similar between the two groups.

## Discussion

In order to identify the challenges and opportunities for a continued decline in TB incidence in Arkansas, we analyzed the incidence trends of culture-confirmed, clustered, and non-clustered TB in different subpopulations, using a combination of TB surveillance data and genotyping results of MTB isolates from culture-confirmed cases reported during January 1, 2004–December 31, 2010. The relative contributions of declines in ongoing TB transmission and declines in reactivation of latent infection were assessed.

The most important finding was that although the incidence of culture-confirmed, clustered, and non-clustered TB declined significantly in Arkansas during 2004–2010, the decline in recent transmission was uneven across different subpopulations. The lack of significant decline in estimated recent transmission among females, non-Hispanic whites, the younger age group (20–64 years), the urban population, and the U.S. born population has an important implication for TB control. Both non-Hispanic whites and females have not been a previously identified risk group for clustering [18], but our data show that TB transmission in these two groups has remained stable. Young age (less than 65 years of age) has previously been identified as a risk factor for being part of large clusters [18]. These data indicate the need for strengthening targeted TB control in subpopulations previously identified as risk groups, but also in subpopulations not previously identified as risk groups.

A second important finding was that the incidence of TB due to reactivation declined similarly among different age, gender, race/ethnicity groups and residence types, as measured by average annual percent decline of incidence. The lower incidence in the younger age group is likely due to the previously described cohort effect where each successive birth cohort has a lower risk of exposure to TB due to the decreasing incidence. As the older members, who have the highest prevalence of latent infection, are removed from the population, the incidence of reactivation of latent infection continues to decrease [5], [10]. The foreign born population had the highest incidence of non-clustered TB throughout the time period and the lack of significant decline represents another target for better control. However, it is important to note that the confidence intervals for the estimated rate of decline among foreign born individualsare wide as the number of foreign born individuals in Arkansas is relatively small (range of foreign born population: 101,169 to 131,667; range of US born population: 2,701,431 to 2,771,280). This relatively small sample size somewhat limits the power to examine the effect of country of origin.

One possible limitation of this study is the use of 12-locus MIRU instead of 24-locus MIRU-VNTR in the cluster definition. This approach might have overestimated the amount of clustering as the 24-locus typing method shows greater discriminatory power than 12-locus method [30]–[32]. The concern about overestimation of clustering may have been offset by the time restriction used in identifying clustered cases in this study. Time-restricted clusters are considered more specific for indicating recent transmission compared to just clustering alone [10]. Furthermore, this study looked at trends over time and any overestimate is expected to be consistent over time. Another potential limitation of this study is the significantly different distributions of age and disease sites between genotyped cases (the study sample) and culture-confirmed cases that were not genotyped. However, given that the study sample included as high as 87% of all culture-confirmed cases, the findings are informative for the development of an improved strategy for TB transmission control in Arkansas. Another possible limitation of the study is some cases classified as non-clustered may theoretically be index cases from other states, which may have overestimated the number of non-clustered cases.

Despite these possible limitations, the lessons learned from this study have important implications for the future of TB control in Arkansas. The significant declines are encouraging signs for control of this disease in Arkansas. However, for greater success in the future, more resources should be direct towards controlling recent transmission. The findings of the study suggest that the Arkansas tuberculosis control program must target both traditional and non-traditional risk groups for successful tuberculosis elimination. Although the implication of the current study findings for TB control seems to be limited to Arkansas, the present study demonstrates that a thorough analysis of TB trends in different population subgroups of a given geographic region or state can lead to a better understanding of the dynamics of TB in this geographic region or state and the identification of non-traditional risk factors for TB transmission. Similar studies in other low incidence populations would provide beneficial data for how to control and eventually eliminate TB in the U.S.

## References

[1] Centers for Disease Control and Prevention (2010). Reported tuberculosis in the United States, 2010. Available: http://www.cdc.gov/tb/statistics/reports/2010/pdf/report2010.pdf. Accessed 25 August 2012.

[2] Centers for Disease Control and Prevention (2012) Trends in tuberculosis–United States, 2011. MMWR 61 (11) 181–5. 22437911

[3] Taylor Z, Nolan CM, Blumberg HM (2005) American Thoracic Society, Centers for Disease Control and Prevention, (2005) et al Controlling tuberculosis in the United States. Recommendations from the American Thoracic Society, CDC, and the Infectious Diseases Society of America. MMWR. Recommendations and reports: Morbidity and mortality weekly report. Recommendations and reports/Centers for Disease Control 54 (RR-12) 1–81. 16267499

[4] Burzynski J, Schluger NW (2008) The epidemiology of tuberculosis in the United States. SeminRespirCrit Care Med 29 (5) 492–498. 10.1055/s-0028-1085701 18810683

[5] Borgdorff M, van der Werf M, de Haas P, Kremer K, van Soolingen D, et al (2005) Tuberculosis elimination in the Netherlands. Emerg Infect Dis 11 (4) 597–602. 15829200 10.3201/eid1104.041103 PMC3320334

[6] Nguyen L, Gilbert G, Marks G (2004) Moleculaūr epidemiology of tuberculosis and recent developments in understanding the epidemiology of tuberculosis. Respirology 9 (3) 313–319. 15363001 10.1111/j.1440-1843.2004.00603.x

[7] Ghosh S, Moonan PK, Cowan L, Granta J, Kammerer S, et al (2012) Tuberculosis Genotyping Information Management System: Enhancing Tuberculosis Surveillance in the United States. Infection, Genetics and Evolution 12 (4) 782–788. 10.1016/j.meegid.2011.10.013 22044522

[8] Braden CR, Templeton GL, Cave MD, Valway S, Onorato IM, et al (1997) Interpretation of restriction fragment length polymorphism analysis of *Mycobacterium tuberculosis* isolates from a state with a large rural population. J Infect Dis Jun;175 (6) 1446–1452. 10.1086/516478 9180185

[9] National TB Controllers Association/CDC Advisory Group on Tuberculosis Genotyping (2004). Guide to the Application of Genotyping to Tuberculosis Prevention and Control. Available: http://www.cdc.gov/tb/programs/genotyping/manual.htm. Accessed 20 September 2012.

[10] France AM, Cave MD, Bates JH, Foxman B, Chu T, et al (2007) What's driving the decline in tuberculosis in Arkansas? A molecular epidemiologic analysis of tuberculosis trends in a rural, low-incidence population, 1997–2003. Am J Epidemiol 166 (6) 662–671. 17625223 10.1093/aje/kwm135

[11] Dillaha J, Yang Z, Ijaz K, Eisenach K, Cave MD, et al (2002) Transmission of *Mycobacterium tuberculosis* in a rural community, Arkansas, 1945–2000. Emerg Infect Dis 8 (11) 1246–1248. 12453349 10.3201/eid0811.020299 PMC2738561

[12] Ellis B, Crawford J, Braden C (2002) Molecular epidemiology of tuberculosis in a sentinel surveillance population. Emerg Infect Dis 8 (11) 1197–1209. 12453343 10.3201/eid0811.020403 PMC2738559

[13] Kempf M, Dunlap N, Lok K, Benjamin WH, Keenan N, et al (2005) Long-term molecular analysis of tuberculosis strains in Alabama, a state characterized by a largely indigenous, low-risk population. J Clin Microbiol 43 (2) 870–878. 15695694 10.1128/JCM.43.2.870-878.2005 PMC548052

[14] Centers for Disease Control and Prevention (2004). Reported tuberculosis in the United States, 2003. http://www.cdc.gov/tb/statistics/reports/2003/pdf/report2003.pdf Accessed 22 September 2012.

[15] Centers for Disease Control and Prevention (CDC). Reported tuberculosis in the United States, 2008. Available: http://www.cdc.gov/tb/statistics/reports/2008/pdf/report2008.pdf. Accessed 22 September 2012.

[16] Centers for Disease Control and Prevention (CDC). Reported tuberculosis in the United States, 2009. Available: http://www.cdc.gov/tb/statistics/reports/2009/pdf/report2009.pdf. Accessed 22 September 2012.

[17] Pratt RH, Winston CA, Kammerer JS, Armstrong LR (2011) Tuberculosis in Older Adults in the United States, 1993–2008. J Am Geriatr Soc 59 (5) 851–857. 21517786 10.1111/j.1532-5415.2011.03369.x

[18] Talarico S, Ijaz K, Zhang X, Mukasa L, Zhang L, et al (2011) Identification of factors for tuberculosis transmission via an integrated multidisciplinary approach. Tuberculosis 91 (3) 244–249. 21367661 10.1016/j.tube.2011.01.007 PMC3142560

[19] Oren E, Winston CA, Pratt R, Robison V, Narita M (2011) Epidemiology of Urban Tuberculosis in the United States, 2000–2007. Am J Public Health 101 (7) 1256–1263. 21566031 10.2105/AJPH.2010.300030 PMC3110232

[20] Eberhardt M, Pamuk E (2004) The importance of place of residence: Examining health in rural and nonrural areas. Am J Public Health 94 (10) 1682–1686. 15451731 10.2105/ajph.94.10.1682 PMC1448515

[21] O'Donnell MR, Chamblee S, von Reyn CF, Ellerbrock TV, Johnson J, et al (2010) Racial disparities in primary and reactivation tuberculosis in a rural community in the southeastern United States. Int J Tuberc Lung Dis 14 (6) 733–740. 20487612

[22] Cain KP, Haley CA, Armstrong LR, Garman K, Wells C, et al (2007) Tuberculosis among foreign-born persons in the United States - Achieving tuberculosis elimination. Am J RespirCrit Care Med 175 (1) 75–79. 10.1164/rccm.200608-1178OC 17038659

[23] U.S. Census Bureau. 2010 Census Interactive Population Search Arkansas. Available: http://2010.census.gov/2010census/popmap/ipmtext.php?fl=05. Accessed 15 February 2012.

[24] Orszag P (2009) Update of statistical area definitions and guidance on their uses. OMB Bulletin 10 (2) 1–154.

[25] National Center for Health Statistics. Postcensal estimates of the resident population of the United States for July 1, 2000–July 1, 2009, by year, county, age, bridged race, Hispanic origin, and sex (Vintage 2009). Prepared under a collaborative arrangement with the U.S. Census Bureau; released June 20, 2010. Available: www.cdc.gov/nchs/nvss/bridged_race.htm as of July 23, 2010. Accessed 15 February 2012.

[26] U.S. Census Bureau, American Community Survey. 2004–2010. Available: http://factfinder2.census.gov/faces/nav/jsf/pages/index.xhtml. Accessed 15 February 2012.

[27] Jasmer RM, Hahn JA, Small PM, Daley C, Behr M, et al (1999) A molecular epidemiologic analysis of tuberculosis trends in San Francisco,1991–1997. Ann Intern Med 130: 971–978. 10383367 10.7326/0003-4819-130-12-199906150-00004

[28] Buchanan I. Calculating Poisson confidence intervals in Excel. Liverpool, United Kingdom: Centre for Public Health, Liver-pool John Moores University, 2004. Available: http://www.nwpho.org.uk/sadb/Poisson/%20CI%20in%20spreadsheets.pdf. Accessed 20 March 2012.

[29] SAS Institute Inc. SAS Version 9.2. Cary, NC: SAS Institute Inc, 2008

[30] Allix-Béguec C, Fauville-Dufaux M, Supply P (2008) Three-Year Population-Based Evaluation of Standardized Mycobacterial lnterspersed Repetitive-Unit-Variable-Number Tandem-Repeat Typing of *Mycobacterium tuberculosis* . J Clin Microbiol 46 (4) 1398–1406. 18234864 10.1128/JCM.02089-07 PMC2292969

[31] Christianson S, Wolfe J, Orr P, Karlowsky J, Levett P, et al (2010) Evaluation of 24 locus MIRU-VNTR genotyping of *Mycobacterium tuberculosis* isolates in Canada. Tuberculosis 90: 31–38. 20056488 10.1016/j.tube.2009.12.003

[32] Supply P, Lesjean S, Savine E, Kremer K, van Soolingen D, et al (2001) Automated high-throughput genotyping for study of global epidemiology of *Mycobacterium tuberculosis* based on mycobacterial interspersed repetitive units. J Clin Microbiol 39 (10) 3563–3571. 11574573 10.1128/JCM.39.10.3563-3571.2001 PMC88389

